# Supplementary material for: Seasonal, Organ-, and Location-Dependent Variations in the Alkaloid Content of Pachysandra terminalis Investigated by Multivariate Data Analysis of LC-MS Profiles
Source: Plants (Basel). 2025 Oct 3;14(19):3060. doi: 10.3390/plants14193060 (PMC12526008; doi:10.3390/plants14193060)
Supplement: Supplementary file 1 [file plants-14-03060-s001.zip › plants-3787934-SI.pdf]

plants

MDPI

Supplementary Materials

Seasonal, Organ-, and Location-Dependent Variations in the Alkaloid Content of *Pachysandra terminalis* Investigated by Multivariate Data Analysis of LC-MS Profiles

Lizanne Schäfer [1], Jandirk Sendker [1], Thomas J. Schmidt [1,*]

[1] Institute of Pharmaceutical Biology and Phytochemistry (IPBP), University of Münster, PharmaCampus Corrensstraße 48, D-48149 Münster, Germany; LS: l_scha57@uni-muenster.de, JS: sendkej@uni-muenster.de
* Correspondence: thomschm@uni-muenster.de; Tel.: +49-251-833-3378

Academic Editor: Firstname Lastname

Received: date
Revised: date
Accepted: date
Published: date

Citation: To be added by editorial staff during production.

Copyright: © 2025 by the authors. Submitted for possible open access publication under the terms and conditions of the Creative Commons Attribution (CC BY) license (https://creativecommons.org/licenses/by/4.0/).

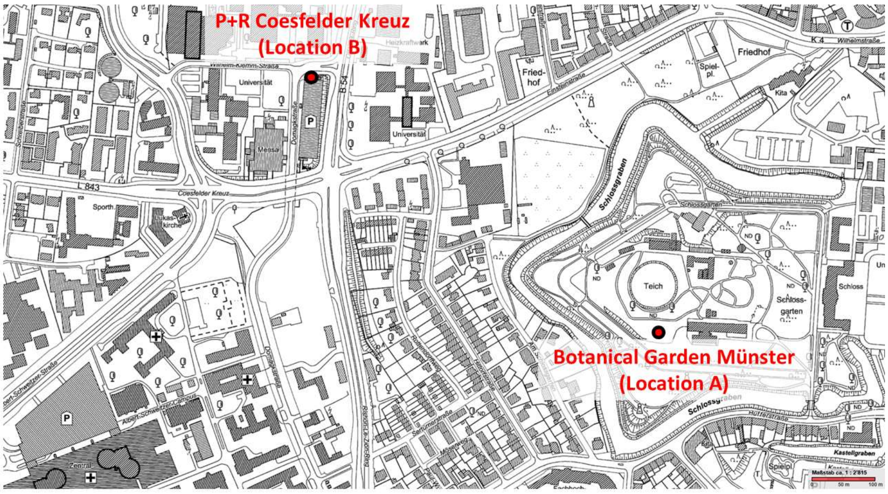

**P+R Coesfelder Kreuz (Location B)**<br>Correnssstraße 28/30<br>48149 Münster, Germany<br>Location of the plant:<br>51.96653° N 7.60115° E	**Botanical Garden Münster (Location A)**<br>Schlossgarten 5<br>48149 Münster, Germany<br>Location of the plant:<br>51.96308° N 7.60919° E

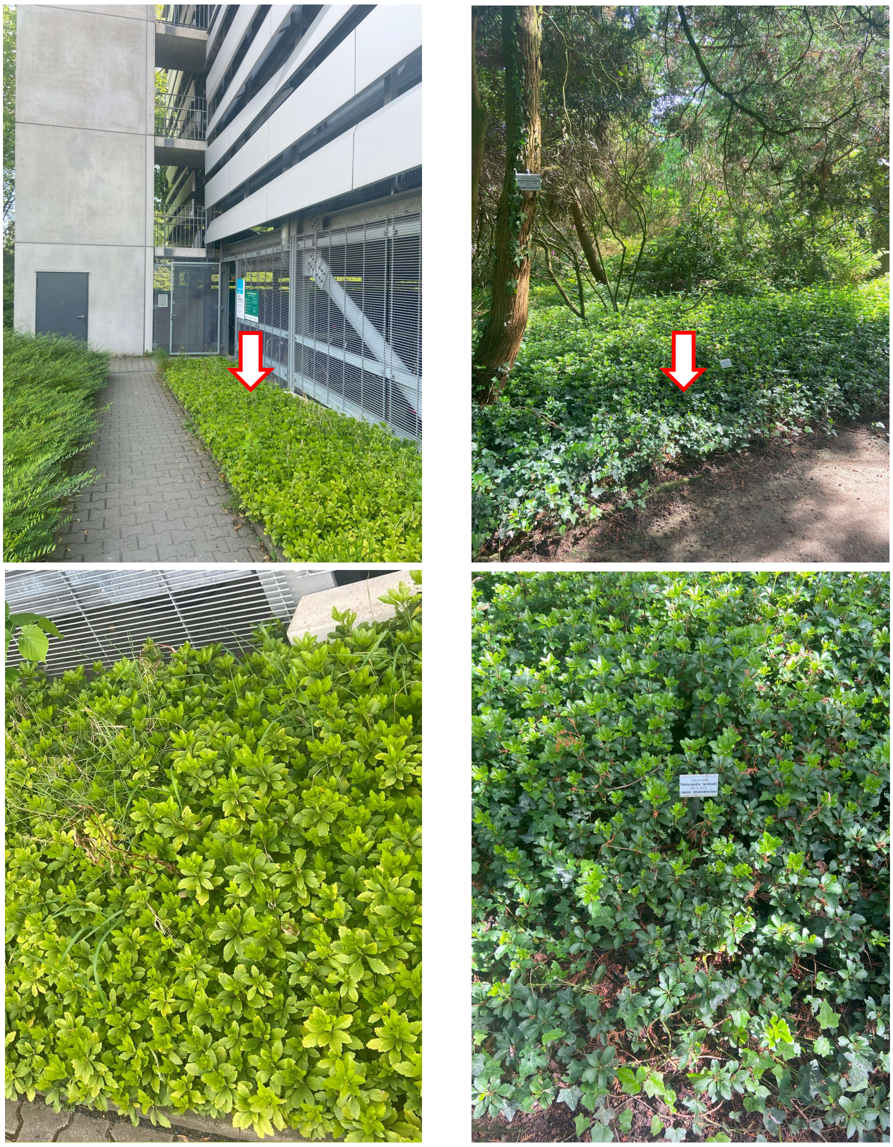

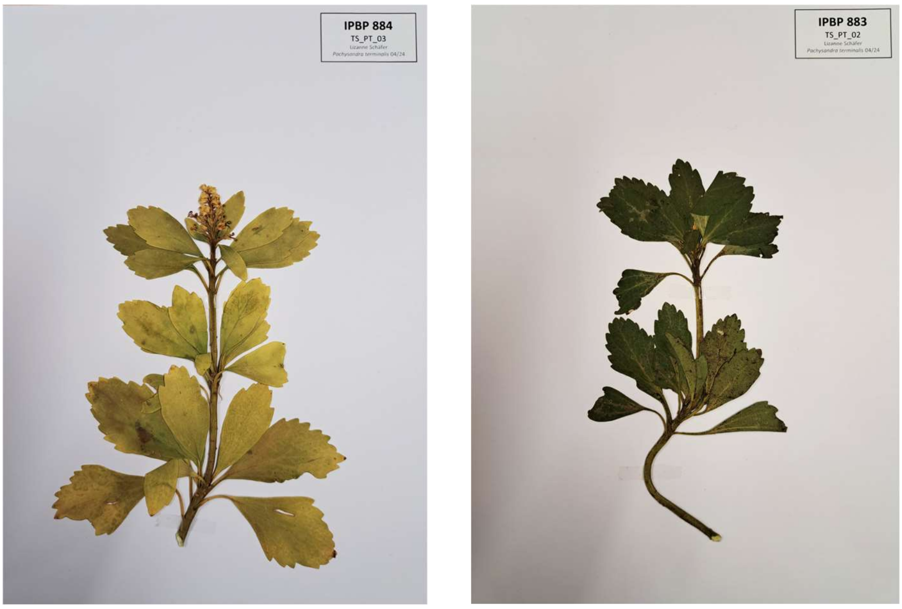

Figure S1. Map and photographs showing the two populations of *Pachysandra terminalis* investigated. Right column: location A, left column: location B. Top: Overall locations of collection; Middle: Populations general view; Bottom: Herbarium specimens of single shoots.

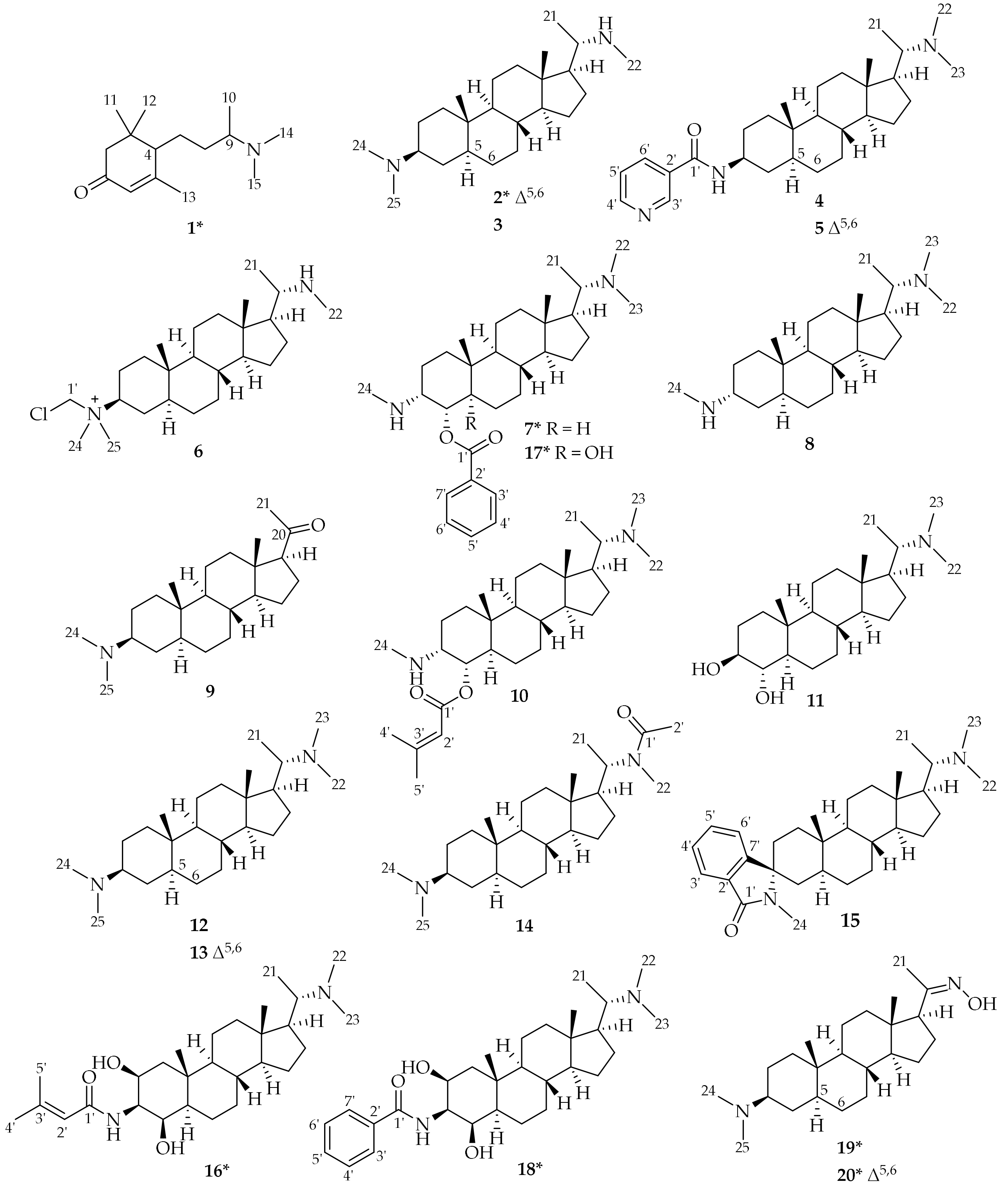

Figure S2. Chemical structures of alkaloids isolated from *Pachysandra terminalis* in our previous study [9]. Compounds **6** and **9** were found to be artefacts formed during the isolation process.

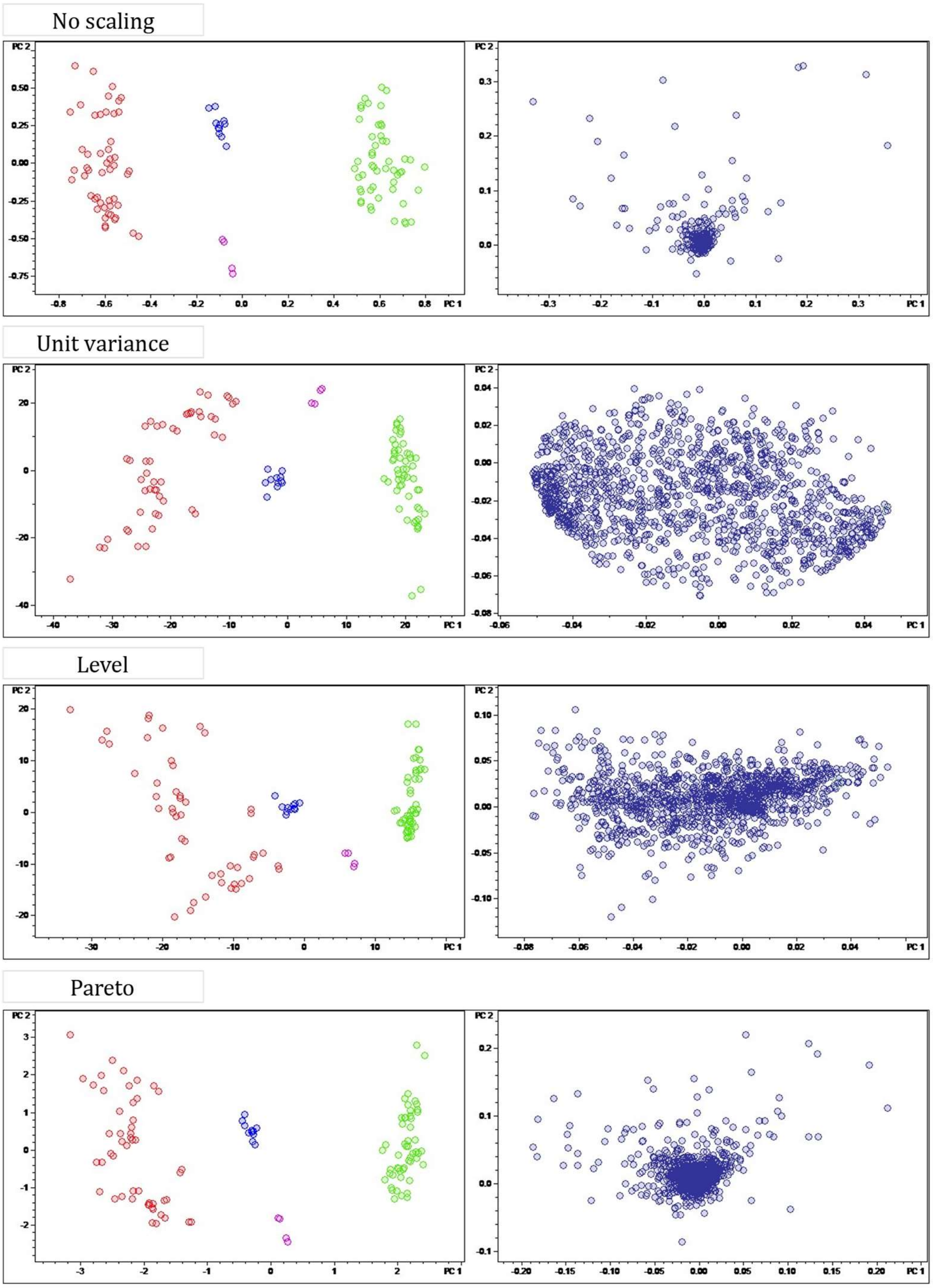

Legend: Plant organs
Red: Leaves, Pink: Flowers, Green: Twigs, Blue: Quality control (Mix)

Figure S3. Principal Component Analysis of the LC/MS data of the seasonal profile from *Pachysandra terminalis* with different scaling methods. Scores plot left, loadings plot right.

Table S1. Characteristic buckets revealed by the principal component analysis and volcano plots of the seasonal profile from *Pachysandra terminalis*.

No.	Bucket	Adduct ions	MS/MS fragments [*m/z*]	Molecular formula	Compound	Characteristic for
1	12.43 min : *m/z* 463.3723	[M+H]$^+$	418 (-45 Da), 228, 172, 136, 105	$C_{31}H_{46}N_2O$	Spiropachysine (**15**)	Leaves
2	8.00 min : *m/z* 403.3704	[M+H]$^+$, [M+2H]$^{2+}$	358, 285, 121, 100	$C_{26}H_{46}N_2O$	Epipachysamine A (**14**)	Botanical Garden
3	2.50 min : *m/z* 238.2163	[M+H]$^+$	151, 123, 109, 95	$C_{15}H_{27}NO$	9-(*N*,*N*-Dimethyl)-5-megastigmen-1-one (**1**)	Botanical Garden
4	12.21 min : *m/z* 467.3666	[M+H]$^+$, [M+2H]$^{2+}$	422, 283, 215, 189, 122	$C_{30}H_{46}N_2O_2$	Epipachysandrine A	Leaves
5	11.71 min : *m/z* 501.4081	[M+H]$^+$	369, 314, 283, 114, 83	$C_{31}H_{52}N_2O_3$	Pachysandrine B	Twigs
6	12.11 min : *m/z* 475.3928	[M+H]$^+$	457 (-18 Da), 328, 281, 114	$C_{29}H_{50}N_2O_3$	Pachystermine C	Location B
7	11.91 min : *m/z* 402.2816	[M+2H]$^{2+}$	281, 157, 105	$C_{28}H_{35}NO$	Aminosteroid+DB+S* oder Aminosteroid+2S*	Leaves
8	12.07 min : m/z 457.3822	[M+H]+	328, 300, 271, 232, 175	$C_{29}H_{48}N_2O_2$	Unknown compound	Twigs
9	4.08 min : m/z 181.1847	[M+H]+, [M+2H]$^{2+}$	316, 285, 175, 161, 135, 121, 95	$C_{24}H_{44}N_2$	Pachysamine A (**8**)	Warm month
10	12.57 min : m/z 465.3863	[M+H]+	418, 285, 172, 136, 107	$C_{29}H_{48}N_2O_2$	Pachysamine H	Warm month
11	12.42 min : m/z 443.4016	[M+H]+, [M+2H]$^{2+}$	398, 285, 191, 114, 83	$C_{28}H_{35}NO$	Pachysamine B	Warm month
12	3.75 min : m/z 377.3536	[M+H]+, [M+2H]$^{2+}$	330, 285, 74	$C_{24}H_{44}N_2O$	Aminosteroid without S or DB*	Cold month
13	11.62 min : m/z 473.3767	[M+H]+	455, 326, 298, 258, 95	$C_{29}H_{48}N_2O_3$	Unknown compound	Cold month
14	11.84 min : *m/z* 523.3931	[M+H]$^+$	418, **283**, 136, 105	$C_{33}H_{50}N_2O_3$	Pachysandrine A/(20S)-20-(Dimethylamino)-3α-(methylbenzoylamino)-5α-pregn-12β-yl acetate	Leaves

No.	Bucket	Adduct ions	MS/MS fragments [*m/z*]	Molecular formula	Compound	Characteristic for
15	12.40 min : *m/z* 485.3764	$[M+H]^+$	83, **281, 298**, 358	$C_{30}H_{48}N_2O_3$	Pachysamine L	Twigs
16	12.39 min : *m/z* 459.3974	$[M+H]^+$	396, 283, 114	$C_{29}H_{50}N_2O_2$	Pachystermine B/(+)-(20S)-20-(di-methylamino)-3α-(methylsene-cioylamino)-5α-pregn-12β-ol	Twigs
17	12.04 min : *m/z* 979.7712	$[M+H]^+$	523, 457	$C_{60}H_{102}N_2O_8$	Unknown compound	Twigs
18	7.00 min : m/z 332.2591	$[M+H]^+$	279, 119, 95	$C_{21}H_{33}NO_2$	Aminosteroid with 2 further S and DB	Location B
19	13.44 min : *m/z* 360.2925	$[M+H]^+$	283	$C_{23}H_{37}NO_2$	Aminosteroid with 1 further S or DB	Location B
20	4.95 min : *m/z* 483.3622	$[M+H]^+$, $[M+2H]^{2+}$	402, 281, 105	$C_{30}H_{46}N_2O_3$	Pachysamine K	Location A
21	6.64 min : *m/z* 226.6883	$[M+H]^+$, $[M+2H]^{2+}$	407, 285, 123	$C_{29}H_{45}N_3O$	Epipachysamine B (**4**)	Location A
22	6.12 min : *m/z* 225.6803	$[M+H]^+$, $[M+2H]^{2+}$	405, **283**, 123	$C_{29}H_{43}N_3O$	Pactermine A (**5**)	Location A
23	6.79 min : *m/z* 463.3913	$[M+H]^+$	418, **285**, 134, 116	$C_{28}H_{50}N_2O_3$	Aminosteroid without further S or DB	Location A
24	4.42 min : *m/z* 432.3587	$[M+H]^+$	415, **285**	$C_{26}H_{45}N_3O_2$	Aminosteroid without further S or DB	Location A
25	14.14 min : m/z 414.3612	$[M+H]^+$	-	$C_{24}H_{47}NO_4$	Unknown compound	Location A

*DB: Double bond of the steroidal skeleton, S: Further substituent besides the substituents at position C-20 and C-3.

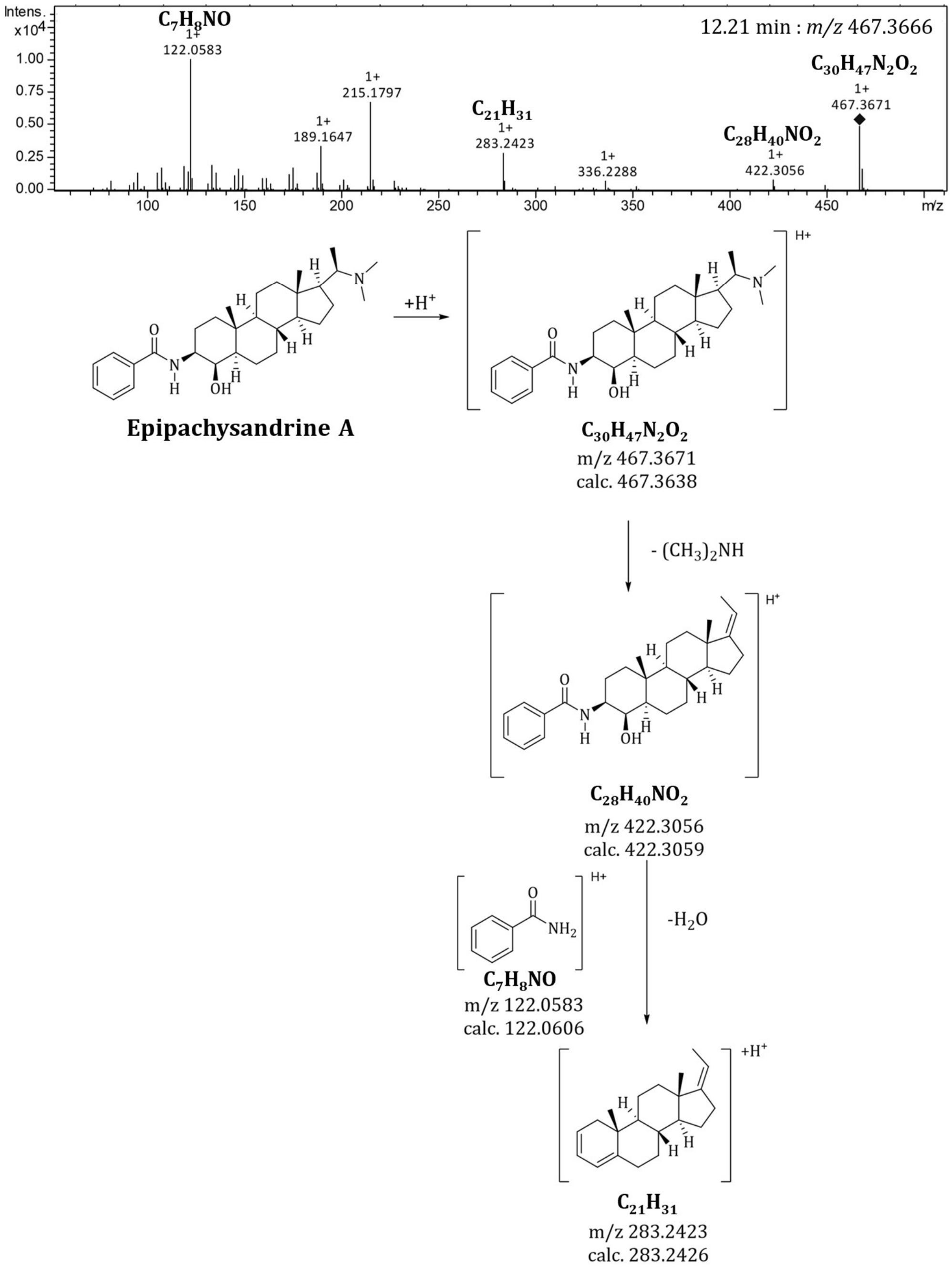

Figure S4. +ESI-QqTOF-MS/MS spectrum of the bucket (12.21 min : *m/z* 467.3666) which was identified as Epipachysandrine A as well as the postulated fragment pattern.

https://doi.org/10.3390/xxxxx

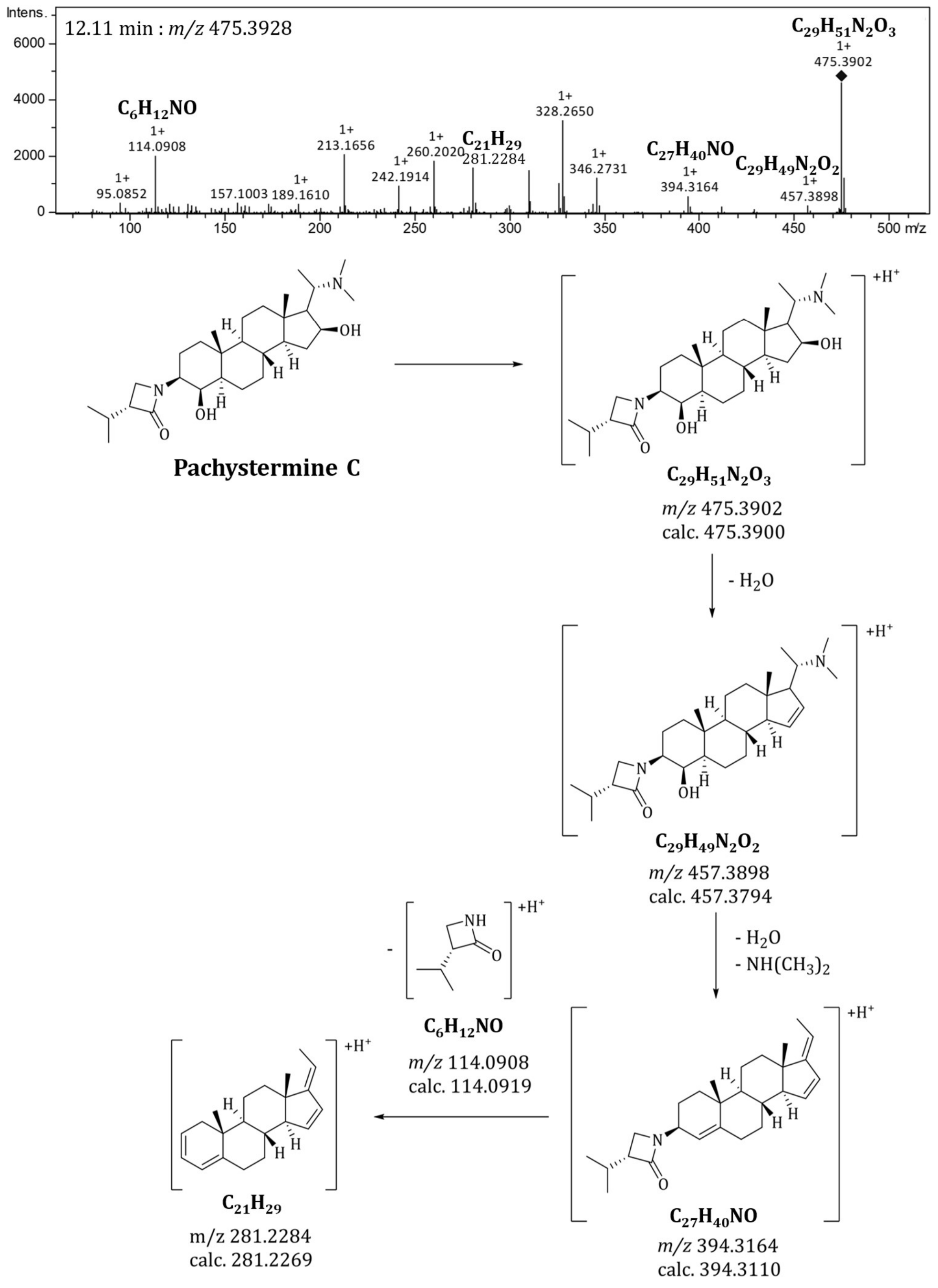

Figure S5. +ESI-QqTOF-MS/MS spectrum of the bucket (12.11 min : *m/z* 475.3928) which was identified as Pachystermine C as well as the postulated fragment pattern.

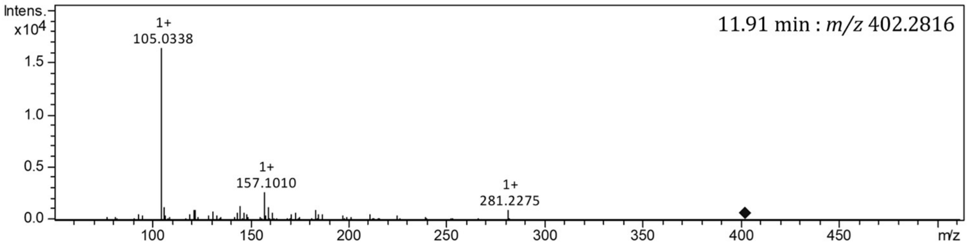

Figure S6. +ESI-QqTOF-MS/MS spectrum of the bucket (11.91 min : *m/z* 402.2816).

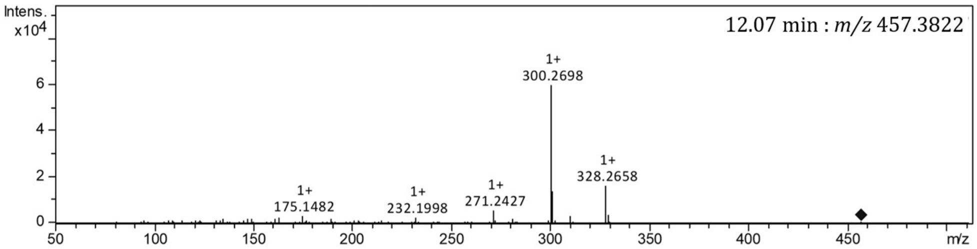

Figure S7. +ESI-QqTOF-MS/MS spectrum of the bucket (12.07 min : *m/z* 457.3822).

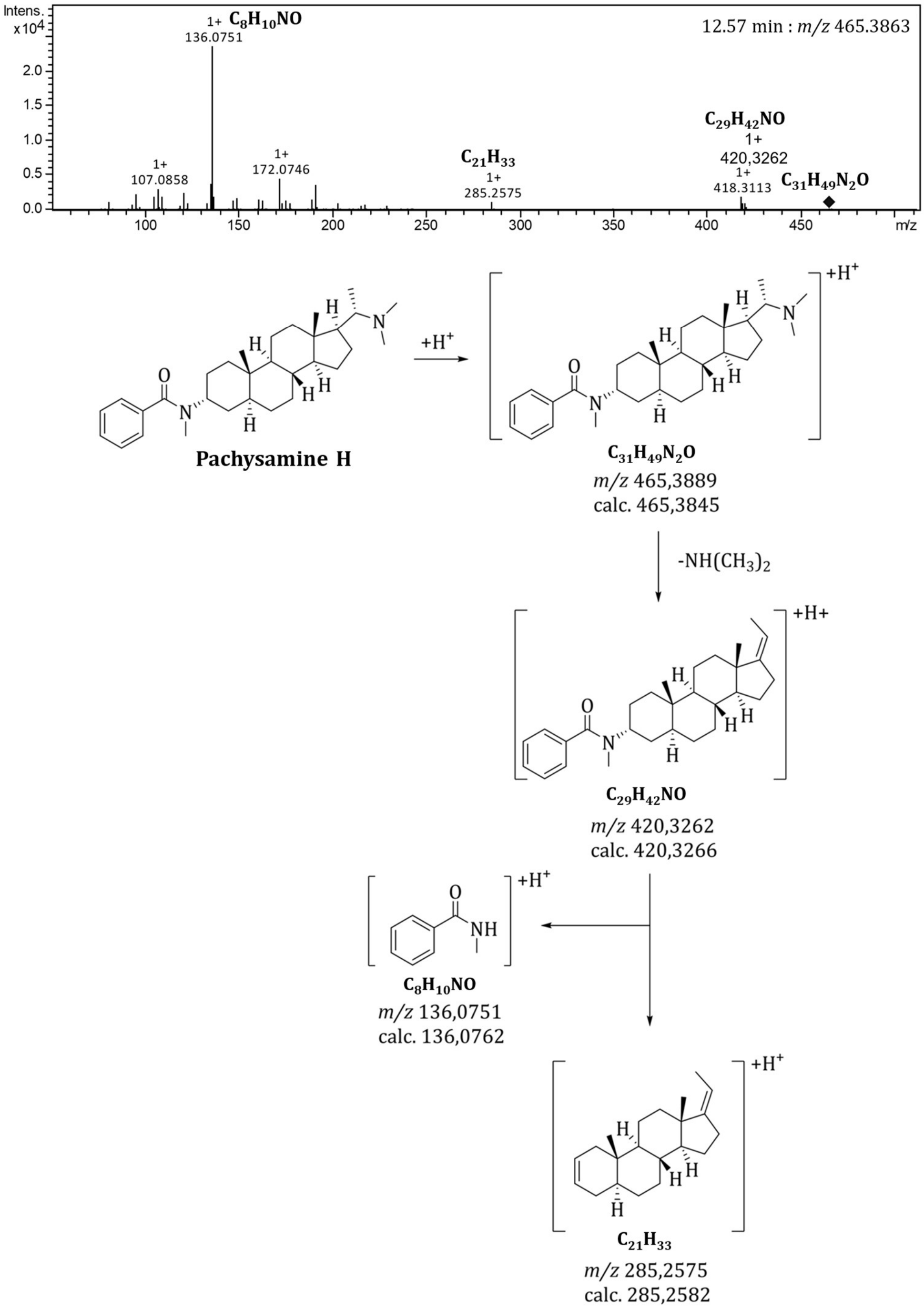

Figure S8. +ESI-QqTOF-MS/MS spectrum of the bucket (12.57 min : *m/z* 465.3863) which was identified as Pachysamine H as well as the postulated fragment pattern.

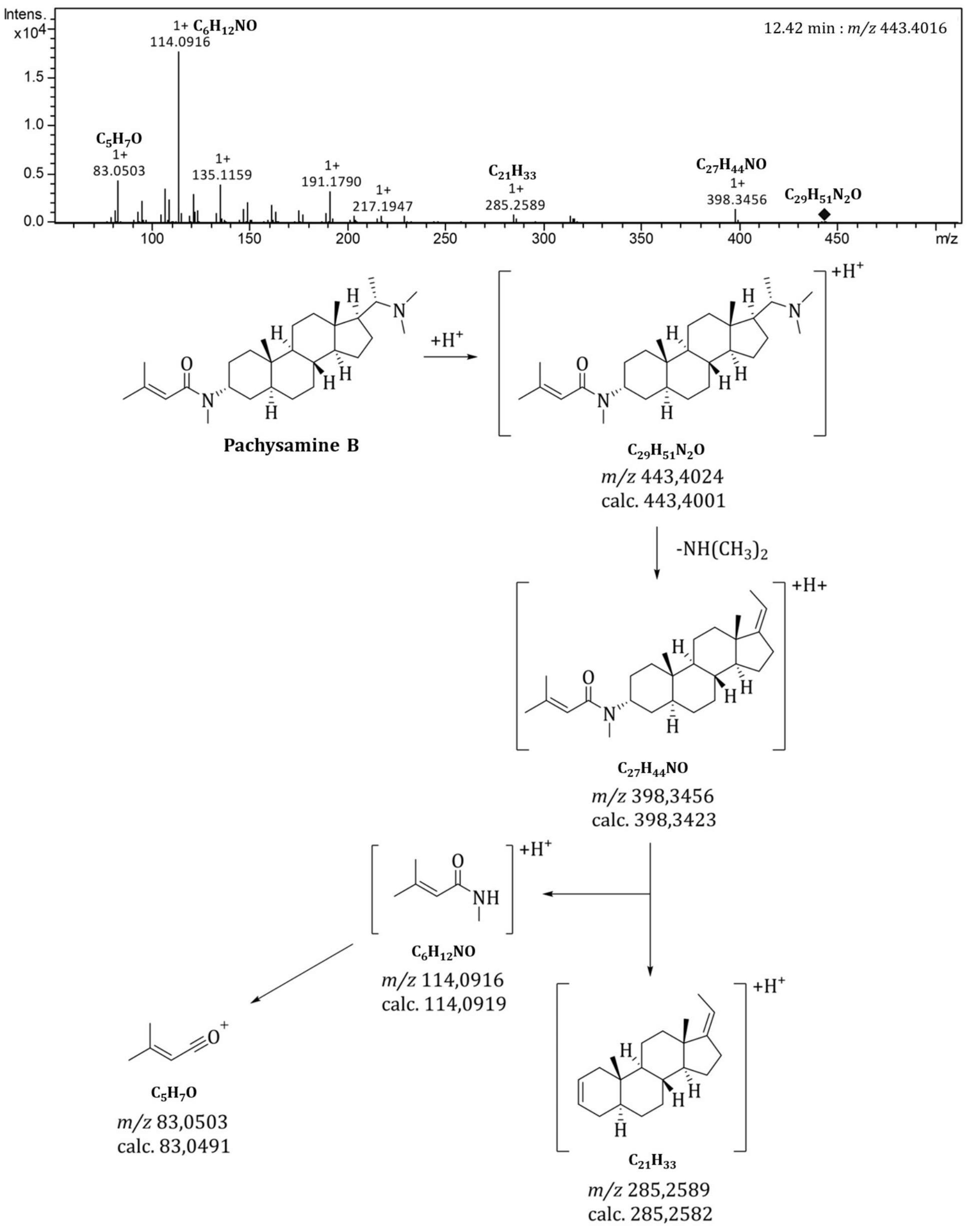

Figure S9. +ESI-QqTOF-MS/MS spectrum of the bucket (12.42 min : *m/z* 443.4016) which was identified as Pachysamine B as well as the postulated fragment pattern.

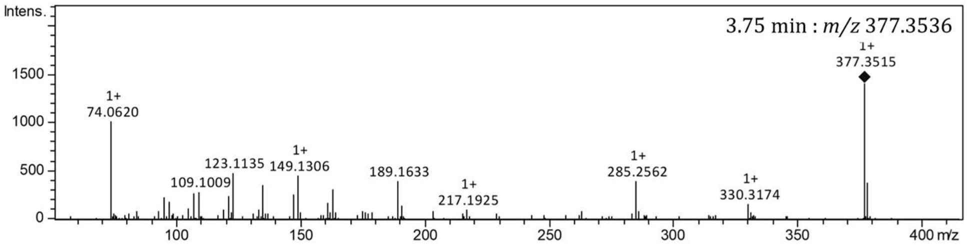

Figure S10. +ESI-QqTOF-MS/MS spectrum of the bucket (3.75 min : *m/z* 377.3536).

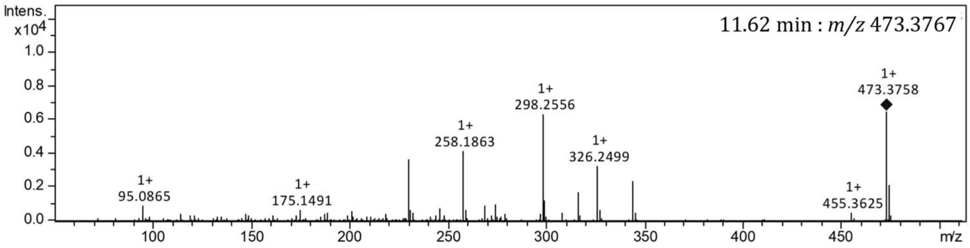

Figure S11. +ESI-QqTOF-MS/MS spectrum of the bucket (11.62 min : *m/z* 473.3767).

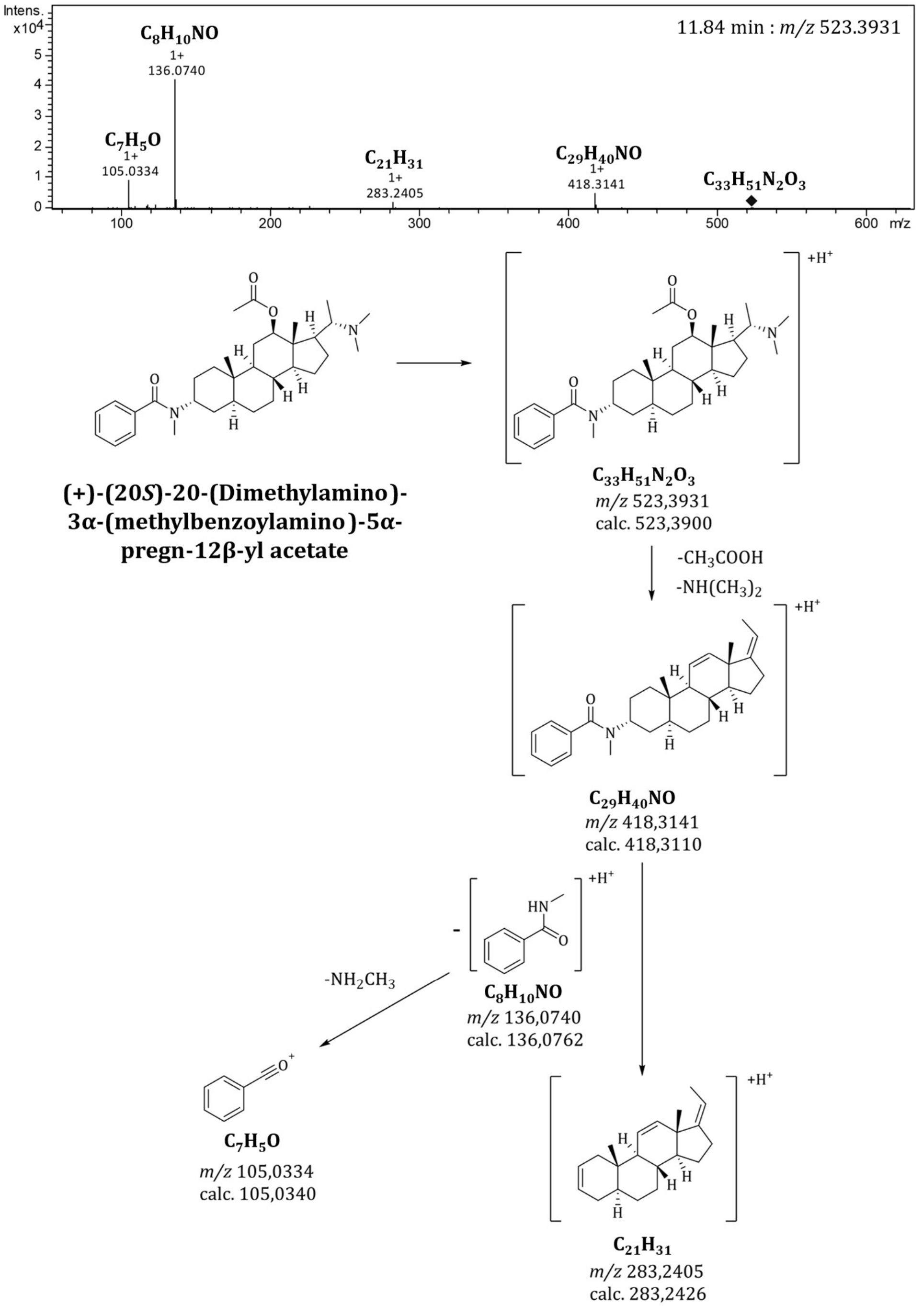

Figure S12. +ESI-QqTOF-MS/MS spectrum of the bucket (11.84 min : m/z 523.3931) which was identified as (+)-(20*S*)-20-(Dimethylamino)-3α-(methylbenzoylamino)-5α-pregn-12β-yl acetate as well as the postulated fragment pattern.

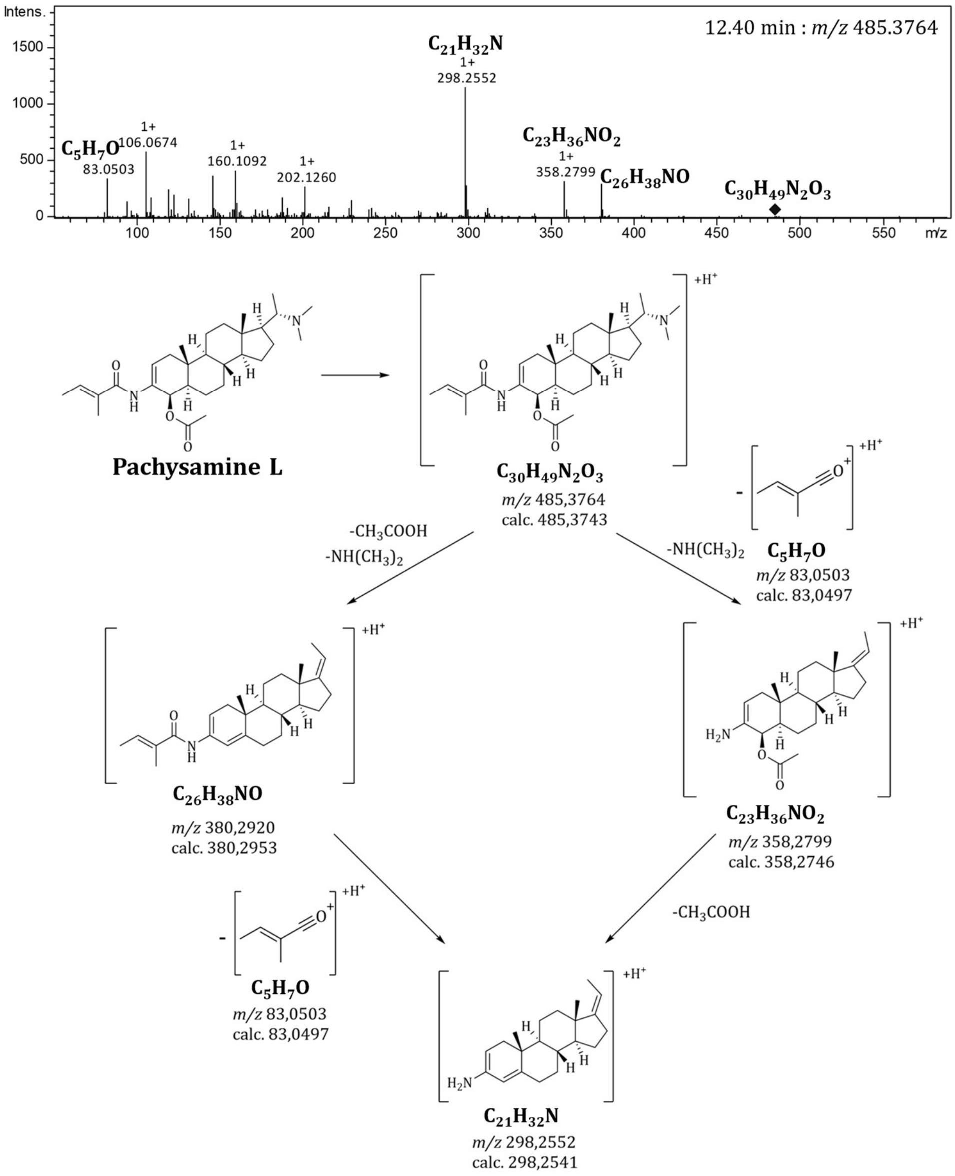

Figure S13. +ESI-QqTOF-MS/MS spectrum of the bucket (12.40 min : m/z 485.3764) which was identified as pachysamine L as well as the postulated fragment pattern.

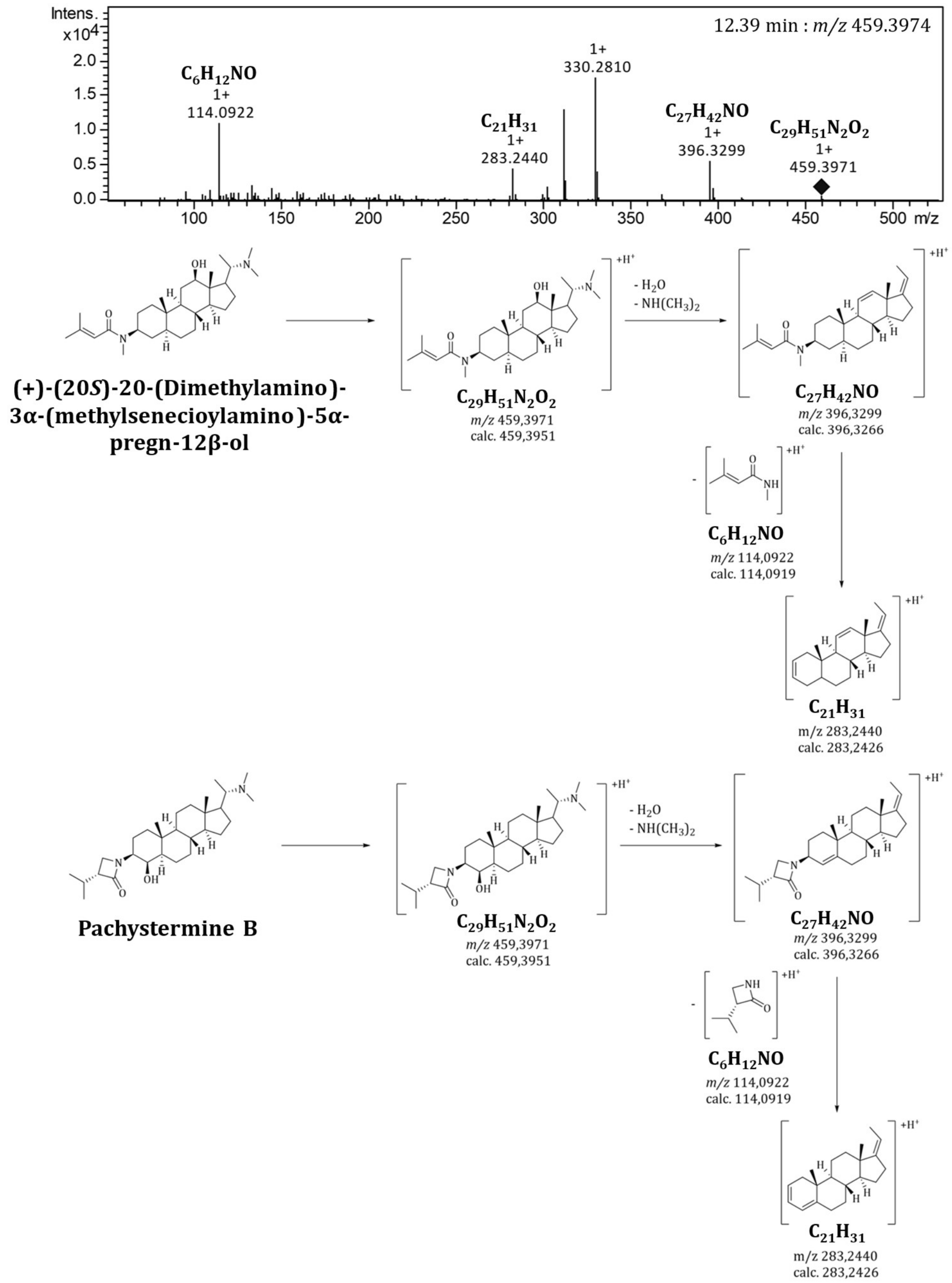

Figure S14. +ESI-QqTOF-MS/MS spectrum of the bucket (12.39 min : m/z 459.3974) which was identified as (+)-(20*S*)-20-(Dimethylamino)-3α-(methylsenecioylamino)-5α-pregn-12β-ol or pachystermine B as well as the postulated fragment pattern.

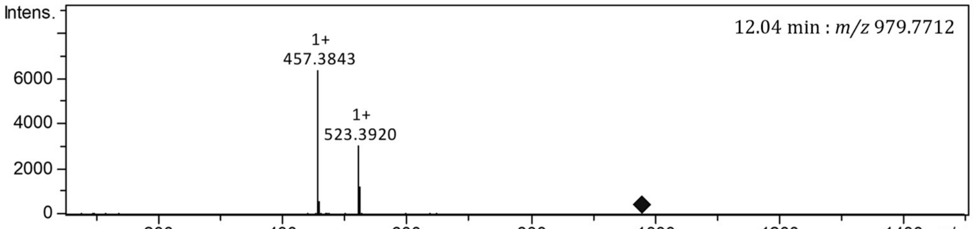

Figure S15. +ESI-QqTOF-MS/MS spectrum of the bucket (12.04 min : *m*/*z* 979.7712).

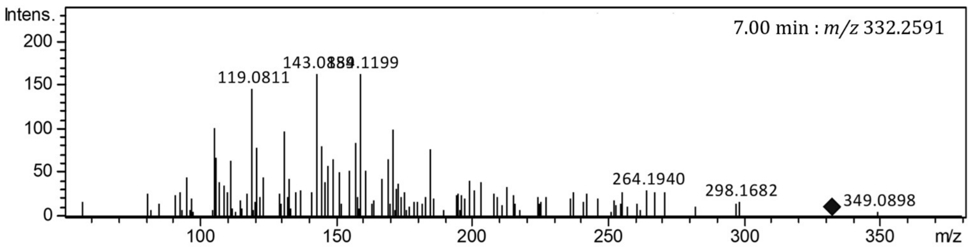

Figure S16. +ESI-QqTOF-MS/MS spectrum of the bucket (7.00 min : *m/z* 332.2591).

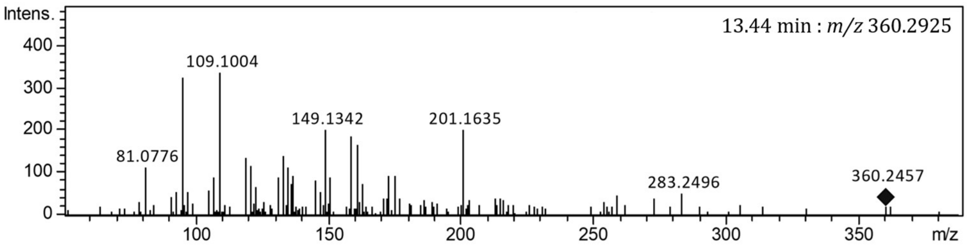

Figure S17. +ESI-QqTOF-MS/MS spectrum of the bucket (13.44 min : *m/z* 360.2925).

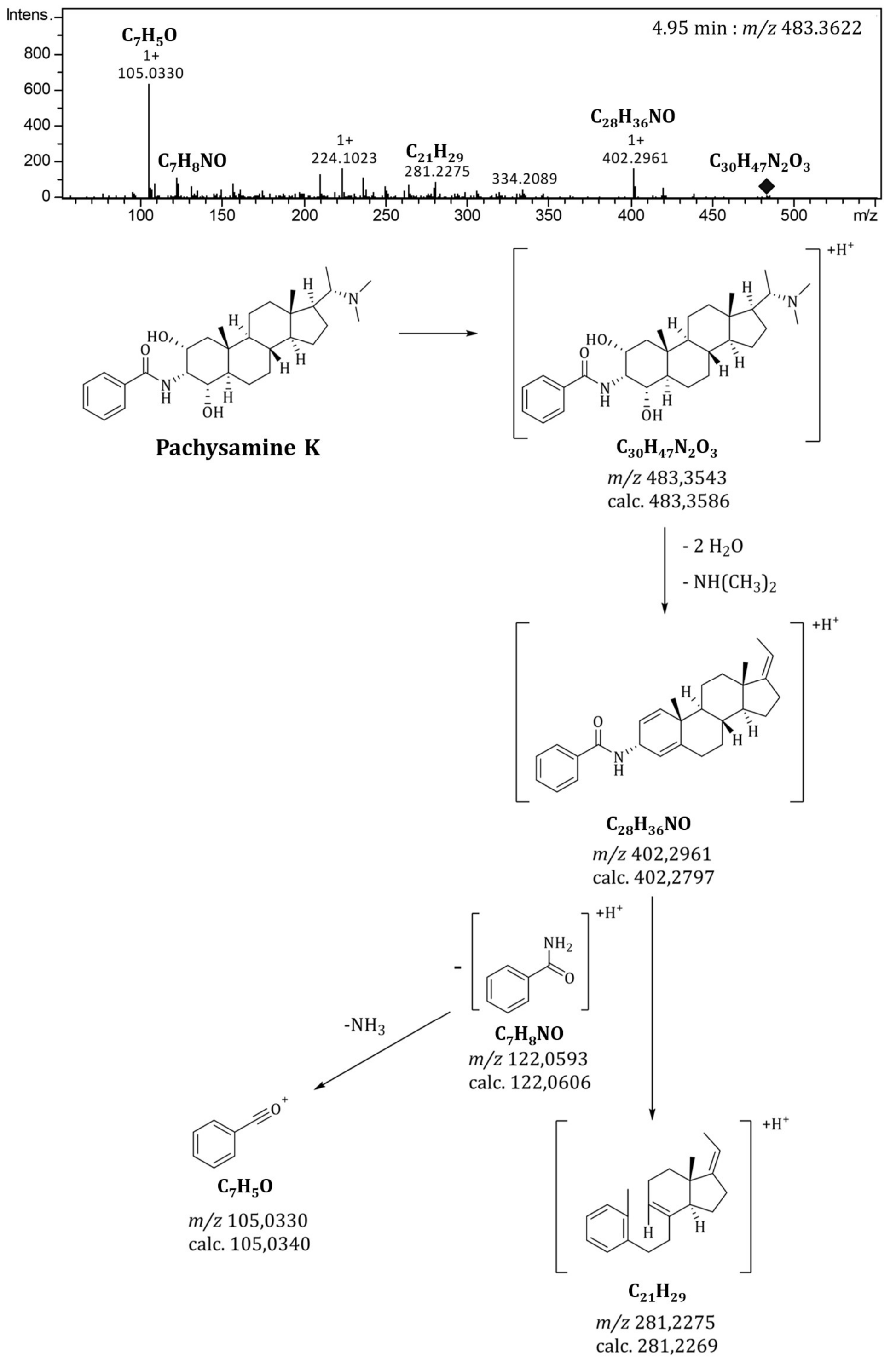

Figure S18. +ESI-QqTOF-MS/MS spectrum of the bucket (4.95 min : m/z 483.3622) which was identified as pachysamine K as well as the postulated fragment pattern.

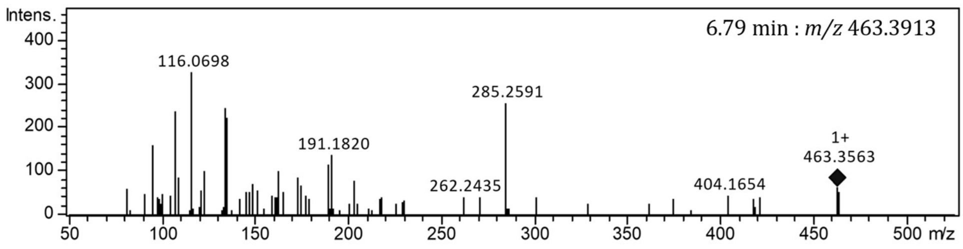

Figure S19. +ESI-QqTOF-MS/MS spectrum of the bucket (6.79 min : *m/z* 463.3913).

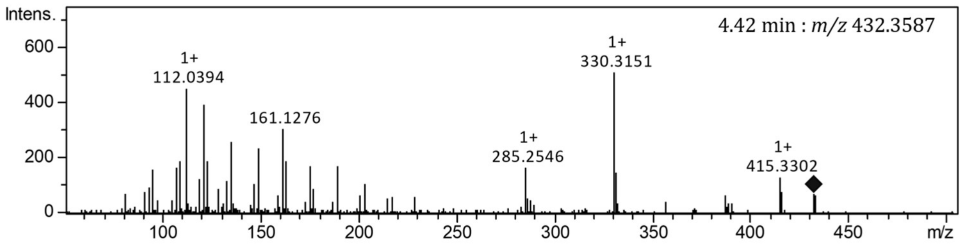

Figure S20. +ESI-QqTOF-MS/MS spectrum of the bucket (4.42 min : *m/z* 432.3587).

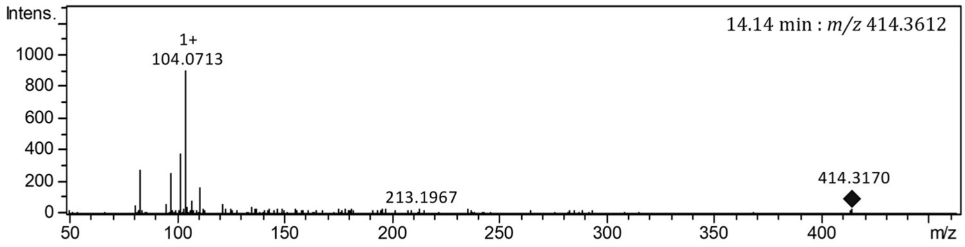

Figure S21. +ESI-QqTOF-MS/MS spectrum of the bucket (14.14 min : *m/z* 414.3612).
